# West of England Sanatorium

**Published:** 1890-03-22

**Authors:** 


### The Hospital Workshop.

(BY OUR SPECIAL COMMISSIONER.)

### No. XXIII.—WEST OF ENGLAND SANATORIUM.

On the North of Somerset the coast is deeply indented between two points, Worle Hill, an ancient British camp, and Bream Down, the proposed site of a harbour for transatlantic ships. Weston-Super-Mare faces the bay thus formed, and somewhere about the middle of its horseshoe, roughly speaking, stands the West of England Sanatorium, a charity that does much excellent and useful work. Its foundation dates from 1868, when twelve patients were accommodated in an old wayside inn bought for the purpose. One large wing was added in 1872, and another in 1882. Upon your commissioner presenting his card one February afternoon, he was courteously received and afforded every information by the Lady Superintendent, Miss Wilson.

The front of the building looks inland, a wise arrangement that gives the living rooms at the back the full benefit of a pleasant seaward view. The entrance hall is lofty and spacious, lighted with handsome windows of stained glass, the work of Messrs. Bell and Co. of Bristol. These windows, and most of the ornamental articles about the place are special gifts of various friends. This hall, it may be said, greets the visitor at once with a sense of the cheerful brightness and perfect taste that pervades the whole building. To the left is the chapel, which has a finely-decorated interior; near the choir are a number of broad-cushioned seats where the crippled are able to lie at full length during service. The body of the chapel is occupied by patients, and the gallery is open to visitors, a privilege of which many avail themselves, especially in the summer. The male wing of the Sanatorium lies on one side and the female on the other. The windows of the living rooms overlooking the sea are large and low, so that those inside can enjoy the view, even when lying or sitting. There are reading and recreation rooms for men and for women, provided with books and various games, while the men have, in addition, a billiard-bagatelle board. An indescribable air of warmth and comfort surrounds this part of the place, and on every hand are couches, easy chairs, and cushioned seats. Each wing has a wide corridor of glass, that must be a useful shelter in wet weather. Passing on to the wards, one is struck by their brightness, which seems to be in a great measure due to the pale tints of the wall and the white coverlets. At one end of the room is a long row of white jugs and basins, ranged along a marble slab, which in turn rests upon a pitch-pine framework. Each patient has his own washing apparatus, and a separate mirror by the side of the bed, as well as a drawer which holds just enough for immediate requirements, the rest of his belongings being kept in a cloak-room. The wards are of various sizes; some have twelve beds, others four to six; while one large dormitory under the roof holds sixteen. The latter is used only in summer, as it is too cold for winter, a defect which will, however, be remedied when the new system of heating is added. One ward is named after the Freemasons, who gave the fittings. There are a few single rooms, for which an extra payment of 3s. a week is required.

Patients are supposed to be well and strong enough to take their meals at the common table, to dress themselves, and to attend to their own wants. Inadmisable cases are persons subject to fits, bedridden, of unsound mind, anyone suffering from advanced phthisis, cancer, infectious or contagious disease, or any malady requiring active medical or surgical treatment. Admission is by subscriber's note, which admits a patient for a fortnight on payment of five shillings a week from April 1st to September 30th, and half-a-crown a week from October 1st to March 31st. Persons not having notes may still obtain admission by a payment of eighteen shillings a-week. During the year 1888, patients' payments amounted to £663 17s. 6d., so that readers will observe that the pay system is in full force in this institution. Any subscriber may have the exclusive use of one bed for a year in return for a donation of £30, for which sum twenty-six "fortnightly" notes are given. In 1888 these "special contributions," as they are called, amounted to £474. There are 100 beds, and during the year mentioned 1,199 persons were admitted. There is a much greater demand for admission during the summer than the winter, although the mild yet bracing air of Weston renders it a valuable wintering place for invalids.

One notable addition to the resources of the establishment has been made lately, in the shape of accommodation for hot and cold sea-water bathing. Two large swimming baths have been erected, one for the men and the other for the women, besides a number of plunge baths, all fitted out in first-rate style. The water is pumped up from the sea into tanks, where the Channel mud is allowed to settle. It is then driven through a Torrens filter and into the baths by a pulsometer patent pump. It may be pretty safely asserted that no other charity in Britain can boast such a means of treatment, which is specially valuable in the class of cases sent to the Sanatorium. The total cost will be about £3,500. An anonymous donor, "Infirmus," gave £1,000, and a large sum has been collected by the Lady Superintendent, but another £1,000 is still wanting.

There is a visiting medical officer, a resident practitioner in the town, and one trained nurse, with an occasional lady probationer. That purely medical treatment is not the most prominent feature is evident from the fact that the drug bill for 1888 was not more than £8 4s. 5d. On the afternoon of our visit the greater number of the patients were out enjoying the bright sun of a lovely February day. A return of patients' occupations gives 375 male artisans and labourers out of 596; five are teachers, and twenty-three clerks. Of the women 285 out of 601 are servants, a somewhat striking fact. Children for 1888 number 88. There is an investment fund of £1,100 for that year. The rules appear to have been carefully drawn up. Number 34, in the light of recent cases in the Law Courts, is one that might be very generally adopted with advantage. It runs: "Alteration of General Rules:—No new rule shall be added, nor shall any rule be amended or rescinded, unless at an Annual or Special General Meeting of Governors, duly convened."

